# From invasion to extinction in heterogeneous neural fields

**DOI:** 10.1186/2190-8567-2-6

**Published:** 2012-03-26

**Authors:** Paul C Bressloff

**Affiliations:** 1 Department of Mathematics, University of Utah, Salt Lake City, UT, 84112, USA

**Keywords:** neural fields, invasive pulled fronts, heterogeneous media, Hamilton-Jacobi equation, path integrals, multiplicative noise, large fluctuations, population extinction.

## Abstract

In this paper, we analyze the invasion and extinction of activity in heterogeneous neural fields. We first consider the effects of spatial heterogeneities on the propagation of an invasive activity front. In contrast to previous studies of front propagation in neural media, we assume that the front propagates into an unstable rather than a metastable zero-activity state. For sufficiently localized initial conditions, the asymptotic velocity of the resulting pulled front is given by the linear spreading velocity, which is determined by linearizing about the unstable state within the leading edge of the front. One of the characteristic features of these so-called pulled fronts is their sensitivity to perturbations inside the leading edge. This means that standard perturbation methods for studying the effects of spatial heterogeneities or external noise fluctuations break down. We show how to extend a partial differential equation method for analyzing pulled fronts in slowly modulated environments to the case of neural fields with slowly modulated synaptic weights. The basic idea is to rescale space and time so that the front becomes a sharp interface whose location can be determined by solving a corresponding local Hamilton-Jacobi equation. We use steepest descents to derive the Hamilton-Jacobi equation from the original nonlocal neural field equation. In the case of weak synaptic heterogenities, we then use perturbation theory to solve the corresponding Hamilton equations and thus determine the time-dependent wave speed. In the second part of the paper, we investigate how time-dependent heterogenities in the form of extrinsic multiplicative noise can induce rare noise-driven transitions to the zero-activity state, which now acts as an absorbing state signaling the extinction of all activity. In this case, the most probable path to extinction can be obtained by solving the classical equations of motion that dominate a path integral representation of the stochastic neural field in the weak noise limit. These equations take the form of nonlocal Hamilton equations in an infinite-dimensional phase space.

## 1 Introduction

Reaction-diffusion equations based on the Fisher-Kolmogorov-Petrovskii-Piskunov (F-KPP) model and its generalizations have been used extensively to describe the spatial spread of invading species including plants, insects, diseases, and genes in terms of propagating fronts [1-7]. One fundamental result in the theory of deterministic fronts is the difference between fronts propagating into a linearly unstable (zero) state and those propagating into a metastable state (a state that is linearly stable but nonlinearly unstable). In the latter case, the front has a unique velocity that is obtained by solving the associated partial differential equation (PDE) in traveling wave coordinates. The former, on the other hand, supports a continuum of possible velocities and associated traveling wave solutions; the particular velocity selected depends on the initial conditions. Fronts propagating into unstable states can be further partitioned into two broad categories: the so-called *pulled *and *pushed *fronts [8] emerging from sufficiently localized initial conditions. Pulled fronts propagate into an unstable state such that the asymptotic velocity is given by the linear spreading speed υ*, which is determined by linearizing about the unstable state within the leading edge of the front. That is, perturbations around the unstable state within the leading edge grow and spread with speed υ*, thus 'pulling along' the rest of the front. On the other hand, pushed fronts propagate into an unstable state with a speed greater than υ*, and it is the nonlinear growth within the region behind the leading edge that pushes the front speeds to higher values. One of the characteristic features of pulled fronts is their sensitivity to perturbations in the leading edge of the wave [9]. This means that standard perturbation methods for studying the effects of spatial heterogeneities [10] or external noise fluctuations [11] break down.

Nevertheless, a number of analytical and numerical methods have been developed to study propagating invasive fronts in heterogeneous media. Heterogeneity is often incorporated by assuming that the diffusion coefficient and the growth rate of a population are periodically varying functions of space. One of the simplest examples of a single population model in a periodic environment was proposed by Shigesada et al. [5,12], in which two different homogeneous patches are arranged alternately in one-dimensional space so that the diffusion coefficient and the growth rate are given by periodic step functions. The authors showed how an invading population starting from a localized perturbation evolves to a traveling periodic wave in the form of a pulsating front. By linearizing around the leading edge of the wave, they also showed how the minimal wave speed of the pulsating front could be estimated by finding solutions of a corresponding Hill equation [12]. The theory of pulsating fronts has also been developed in a more general and rigorous setting [13-15]. An alternative method for analyzing fronts in heterogeneous media, which is applicable to slowly modulated environments, was originally developed by Freidlin [16-18] using large deviation theory and subsequently reformulated in terms of PDEs by Evans and Sougandis [19]. More recently, it has been used to study waves in heterogeneous media (see for example [10,13]). The basic idea is to rescale space and time so that the front becomes a sharp interface whose location can be determined by solving a corresponding Hamilton-Jacobi equation.

Another important topic in population biology is estimating the time to extinction of a population in the presence of weak intrinsic or extrinsic noise sources, after having successfully invaded a given spatial domain [20]. The zero state (which is unstable in the deterministic limit) now acts as an absorbing state, which can be reached via noise-induced transitions from the nontrivial metastable steady state. The most probable path to extinction is determined in terms of classical solutions of an effective Hamiltonian dynamical system [21-24]. The latter can be obtained in the weak noise limit by considering a Wentzel-Kramers-Brillouin (WKB) approximation of solutions to a master equation (intrinsic noise) or Fokker-Planck equation (extrinsic noise) [25-27]; alternatively, the Hamilton equations can be obtained from a corresponding path integral representation of the stochastic population model [21].

In this paper, we extend the Hamiltonian-based approaches to invasion and extinction in reaction-diffusion models to the case of a scalar neural field model. Neural fields represent the large-scale dynamics of spatially structured networks of neurons in terms of nonlinear integrodifferential equations, whose associated kernels represent the spatial distribution of neuronal synaptic connections. Such models provide an important example of spatially extended dynamical systems with nonlocal interactions. As in the case of reaction diffusion systems, neural fields can exhibit a rich repertoire of wave phenomena, including solitary traveling fronts, pulses, and spiral waves [28-30]. They have been used to model wave propagation in cortical slices [31,32] and *in vivo *[33]. A common *in vitro *experimental method for studying wave propagation is to remove a slice of brain tissue and bathe it in a pharmacological medium that blocks the effects of inhibition. Synchronized discharges can then be evoked by a weak electrical stimulus to a local site on the slice, and each discharge propagates away from the stimulus at a characteristic speed of about 60 to 90 mm/s [32,34]. These waves typically take the form of traveling pulses with the decay of activity at the trailing edge resulting from some form of local adaptation or refractory process. On the other hand, a number of phenomena in visual perception involve the propagation of a traveling front, in which a suppressed visual percept replaces a dominant percept within the visual field of an observer. A classical example is the wave-like propagation of perceptual dominance during binocular rivalry [35-38].

In the case of a scalar neural field equation with purely excitatory connections and a sigmoidal or Heaviside firing rate function, it can be proven that there exists a traveling front solution with a unique speed that depends on the firing threshold and the range/strength of synaptic weights [39,40]. The wave, thus, has characteristics typical of a front propagating into a metastable state. Various generalizations of such front solutions have also been developed in order to take into account the effects of network in-homogeneities [41-43], external stimuli [44,45], and network competition in a model of binocular rivalry waves [38]. As far as we are aware, however, there has been very little work on neural fields supporting pulled fronts, except for an analysis of pulsating pulled fronts in [42]. One possible motivation for considering such fronts is that they arise naturally in the deterministic limit of stochastic neural fields with a zero absorbing state. Indeed, Buice and Cowan [46] have previously used path integral methods and renormalization group theory to establish that a stochastic neural field with an absorbing state belongs to the universality class of directed percolation models and consequently exhibits power law behavior suggestive of several measurements of spontaneous cortical activity *in vitro *and *in vivo *[47,48].

We begin by considering a neural field model that supports propagating pulled fronts, and determine the asymptotic wave speed by linearizing about the unstable state within the leading edge of the front (Section 2). We then introduce a spatial heterogeneity in the form of a slow modulation in the strength of synaptic connections (Section 3). Using a WKB approximation of the solution of a rescaled version of the neural field equation and carrying out steepest descents, we derive a local Hamilton-Jacobi equation for the dynamics of the sharp interface (in rescaled space-time coordinates). We then use perturbation methods to solve the associated Hamilton equations under the assumption that the amplitude of the spatial modulations is sufficiently small (Section 4). The resulting solution determines the location of the front as a function of time, from which the instantaneous speed of the front can be calculated. In the case of linearly varying modulations, the position of the front is approximately a quadratic function of time. In the second part of the paper, we investigate how time-dependent heterogeneities in the form of extrinsic multiplicative noise can induce rare noise-driven transitions to the zero-activity state, which now acts as an absorbing state signaling the extinction of all activity (on a shorter time scale, noise results in a subdiffusive wandering of the front [49]). We proceed by first constructing a path integral representation of the stochastic neural field (Section 5). The most probable path to extinction can then be obtained by solving the classical equations of motion that dominate the path integral representation in the weak noise limit; these equations take the form of nonlocal Hamilton equations in an infinite-dimensional phase space (Section 6).

## 2 Neural fields with propagating pulled fronts

We begin by constructing a homogeneous neural field equation that supports a propagating pulled front, and calculate its wave speed. We will adopt the activity-based rather than voltage-based representation of a neural field by taking

(2.1) $$\tau \frac{\partial a\left(x,t\right)}{\partial t}=-a\left(x,t\right)+F\left(\int_{-\infty }^{\infty }w\left(x-x^{\prime }\right)a\left(x^{\prime },t\right)dx^{\prime }\right).$$

For the moment, we consider an unbounded domain with *x *∈ ℝ. Here, the field *a*(*x, t*) represents the instantaneous firing rate of a local population of neurons at position *x *and time *t, w*(*x*) is the distribution of synaptic weights, *τ *is a time constant, and *F *is a nonlinear activation function (for a detailed discussion of different neural field representations and their derivations, see the reviews [28,30]). We also have the additional constraint that *a*(*x, t*) *≥ *0 for all (*x, t*). Note that the restriction to positive values of *a *is a feature shared with population models in ecology or evolutionary biology, for example, where the corresponding dependent variables represent number densities. Indeed, Equation 2.1 has certain similarities with a nonlocal version of the F-KPP equation, which takes the form [50]

(2.2) $$\tau \frac{\partial p\left(x,t\right)}{\partial t}=D\frac{\partial^{2}p\left(x,t\right)}{\partial x^{2}}+\mu p\left(x,t\right)\left(1-\int_{-\infty }^{\infty }K\left(x-x^{\prime }\right)p\left(x^{\prime },t\right)dx^{\prime }\right).$$

One major difference from a mathematical perspective is that Equation 2.2 supports traveling fronts even when the range of the interaction kernel *K *goes to zero, that is, *K*(*x*) → *δ*(*x*), since we recover the standard local F-KPP equation [1,2]. In particular, as the nonlocal interactions appear nonlinearly in Equation 2.2, they do not contribute to the linear spreading velocity in the leading edge of the front. On the other hand, nonlocal interactions play a necessary role in the generation of fronts in the neural field equation (Equation 2.1).

Suppose that *F*(*a*) in Equation 2.1 is a positive, bounded, monotonically increasing function of *a *with *F*(0) = 0, lim_*a*→0+ _*F*'(*a*) = 1 and lim_*a*→∞ _*F*(*a*) = *κ *for some positive constant *κ*. For concreteness, we take

(2.3) $$F\left(a\right)=\left\{\begin{array}{cc} 0, & a\leq 0\\ a,\;0 & <\;a\;\leq \;\kappa \\ \kappa , & a\;>\;\kappa . \end{array}\right)\;$$

A homogeneous fixed point solution a* of Equation 2.1 satisfies

(2.4) $$a^{*}=F\left(W_{0}a^{*}\right),\;\;\;W_{0}=\int_{-\infty }^{\infty }w\left(y\right)dy.$$

**Figure 1:**
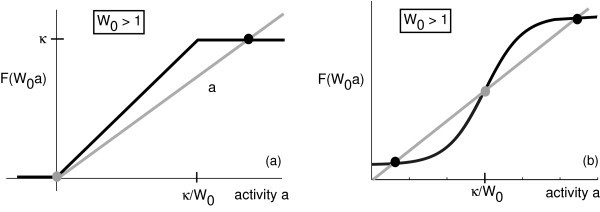
**Plots of firing rate function**. Intercepts of *y *= *F*(*W*_0 _*a*) with a straight line *y *= *a *determine homogeneous fixed points. (**a**) Piecewise linear rate function (Equation 2.3) showing the existence of an unstable fixed point at *a *= 0 and a stable fixed point at *a *= *κ*. (**b**) Sigmoidal rate function *F*(*a*) = 2/(1 + e^-2[*a*-*κ*]^) showing the existence of two stable fixed points separated by an unstable fixed point.

In the case of the given piecewise linear firing rate function, we find that if *W*_0 _*>*1, then there exists an unstable fixed point at a* = 0 (absorbing state) and a stable fixed point at a* = *κ *(see Figure 1a). The construction of a front solution linking the stable and unstable fixed points differs considerably from that considered in neural fields with sigmoidal or Heaviside nonlinearities [28,39], where the front propagates into a metastable state (see Figure 1b). Following the PDE theory of fronts propagating into unstable states [8], we expect that there will be a continuum of front velocities and associated traveling wave solutions. A conceptual framework for studying such solutions is the linear spreading velocity υ*, which is the asymptotic rate with which an initial localized perturbation spreads into an unstable state based on the linear equations obtained by linearizing the full nonlinear equations about the unstable state. Thus, we consider a traveling wave solution A(x-ct) of Equation 2.1 with A(ξ)→κ as *ξ *→ -∞ and A(ξ)→0 as *ξ *→ *∞*. One can determine the range of velocities *c *for which such a solution exists by assuming that $A\left(\xi \right)\approx e^{-\lambda \xi }$ for sufficiently large *ξ*.

The exponential decay of the front suggests that we linearize Equation 2.1, which, in traveling wave coordinates (with *τ *= 1), takes the form

(2.5) $$-c\frac{dA\left(\xi \right)}{d\xi }=-A\left(\xi \right)+\int_{-\infty }^{\infty }w\left(\xi -\xi^{\prime }\right)A\left(\xi^{\prime }\right)d\xi^{\prime }.$$

However, in order to make the substitution $A\left(\xi \right)\approx e^{-\lambda \xi }$, we need to restrict the integration domain of *ξ*' to the leading edge of the front. Suppose, for example, that *w*(*x*) is given by the Gaussian distribution,

(2.6) $$w\left(x\right)=\frac{W_{0}}{\sqrt{2\pi \sigma^{2}}}{\text{e}}^{-x^{2}/2\sigma^{2}}.$$

Given the fact that the front solution A(ξ) is bounded, we introduce a cutoff *X *with *σ *≪ *X *≪ *ξ *and approximate Equation 2.5 by

(2.7) $$-c\frac{dA\left(\xi \right)}{d\xi }=-A\left(\xi \right)+\int_{\xi -X}^{\xi +X}w\left(\xi -\xi^{\prime }\right)A\left(\xi^{\prime }\right)d\xi^{\prime }.$$

Substituting the exponential solution in Equation 2.5 then yields the dispersion relation *c *= *c*(*λ*) with

(2.8) $$c\left(\lambda \right)=\frac{1}{\lambda }\left[\int_{-X}^{X}w\left(y\right){\text{e}}^{-\lambda y}dy-1\right].$$

Finally, we now take the limit *X *→ ∞ under the assumption that *w*(*y*) is an even function to yield

(2.9) $$c\left(\lambda \right)=\frac{1}{\lambda }\left[W\left(\lambda \right)-1\right],$$

where $W\left(\lambda \right)=\hat{W}\left(\lambda \right)+\hat{W}\left(-\lambda \right)$ and $\hat{W}\left(\lambda \right)$ is the Laplace transform of *w*(*x*):

(2.10) $$\hat{W}\left(\lambda \right)=\int_{-\infty }^{\infty }w\left(y\right){\text{e}}^{-\lambda y}dy.$$

We are assuming that *w*(*y*) decays sufficiently fast as *|y| *→ ∞ so that the Laplace transform $\hat{W}\left(\lambda \right)$ exists for bounded, negative values of *λ*. This holds in the case Gaussian distribution (Equation 2.6). In particular,

(2.11) $$\begin{array}{ccc} W\left(\lambda \right) & =\int_{-\infty }^{\infty }w\left(y\right){\text{e}}^{-\lambda y}dy & \\ & =\frac{W_{0}}{\sqrt{2\pi \sigma^{2}}}\int_{-\infty }^{\infty }{\text{e}}^{-y^{2}/2\sigma^{2}}{\text{e}}^{-\lambda y}dy\\ & =W_{0}{\text{e}}^{\lambda^{2}\sigma^{2}/2}. \end{array}$$

Hence,

(2.12) $$c\left(\lambda \right)=\frac{W_{0}{\text{e}}^{\lambda^{2}\sigma^{2}/2}-1}{\lambda }\cdot$$

If *W*_0 _*>*1 (necessary for the zero-activity state to be unstable), then *c*(*λ*) is a positive unimodal function with *c*(*λ*) → ∞ as *λ *→ 0 or *λ *→ ∞ and a unique minimum at *λ *= *λ*_0 _with *λ*_0 _the solution to the implicit

(2.13) $${\lambda_{0}}^{2}=\frac{W_{0}-{\text{e}}^{-{\lambda_{0}}^{2}\sigma^{2}/2}}{\sigma^{2}W_{0}}.$$

**Figure 2:**
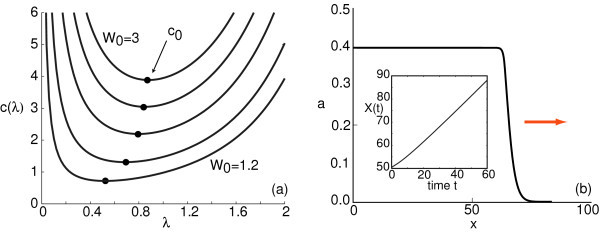
**Velocity dispersion curves and asymptotic front profile**. (**a**) Velocity dispersion curves *c *= *c*(*λ*) for a pulled front solution of the neural field equation (Equation 2.1) with piecewise linear firing rate function (Equation 2.3) and a Gaussian weight distribution (Equation 2.6) with amplitude *W*_0_. Here, *σ *= 1.0, *κ *= 0.4, and *W*_0 _= 1, 2, 1.5, 2.0, 2.5, 3.0. Black dots indicate a minimum wave speed *c*_0 _for each value of *W*_0_. (**b**) Asymptotic front profile in the case *W*_0 _= 1.2. Inset: linear displacement *X*(*t*) of a level set of the front as a function of time *t*.

Example dispersion curves are shown in Figure 2a for various values of the Gaussian weight amplitude *W*_0_. Combining Equations 2.12 and 2.13 shows that

(2.14) $$\frac{c_{0}}{\lambda_{0}}=\sigma^{2}W_{0}{\text{e}}^{{\lambda_{0}}^{2}\sigma^{2}/2}=\sigma^{2}\left(\lambda_{0}c_{0}+1\right),$$

so that

(2.15) $$\lambda_{0}=\frac{1}{2}\left[-\frac{1}{c_{0}}+\sqrt{\frac{1}{{c_{0}}^{2}}+\frac{4}{\sigma^{2}}}\right].$$

Assuming that the full nonlinear system supports a pulled front, then a sufficiently localized initial perturbation (one that decays faster than ${\text{e}}^{-\lambda_{0}x}$) will asymptotically approach the traveling front solution with the minimum wave speed *c*_0 _= *c*(*λ*_0_). Note that *c*_0 _~ *σ *and *λ*_0 _~ *σ*^-1^. In Figure 2b, we show an asymptotic front profile obtained by numerically solving the neural field equation (Equation 2.1) when *W*_0 _= 1.2 (see Section 4.3). The corresponding displacement of the front is a linear function of time with a slope consistent with the minimal wave speed *c*_0 _*≈ *0.7 of the corresponding dispersion curve shown in Figure 2a. This wave speed is independent of *κ*.

In the above analysis, we neglected the effects of boundary conditions on front propagation, which is a reasonable approximation if the size *L *of the spatial domain satisfies *L *≫ *σ*, where *σ *is the range of synaptic weights. In the case of a finite domain, following passage of an invasive activity front, the network settles into a nonzero stable steady state, whose spatial structure will depend on the boundary conditions. The steady-state equation takes the form

(2.16) $$a\left(x\right)=F\left(\int_{0}^{L}w\left(x-x^{\prime }\right)a\left(x^{\prime }\right)dx^{\prime }\right).$$

**Figure 3:**
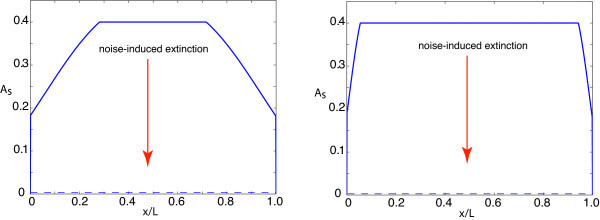
**Stable steady-state solutions**. Stable steady-state solution *a*(*x, t*) = *A_s_*(*x*) of neural field equation (Equation 2.1) on a finite spatial domain of length *L *with boundary conditions *a*(0, *t*) = *a*(*L, t*) = 0. Here, *W*_0 _= 1.2, *σ *= 1, and *κ *= 0.4. (**a**) *L *= 5. (**b**) *L *= 25. In the presence of multiplicative noise, fluctuations can drive the network to the zero absorbing state, resulting in the extinction of activity (see Section 6).

In the case of the Dirichlet boundary conditions, *a*(0, *t*) = *a*(*L, t*) = 0 with *L *≫ *σ*, the steady state will be uniform in the bulk of the domain with *a*(*x*) *≈ a*_0 _except for boundary layers at both ends. Here, *a*_0 _is the nonzero solution to the equation *a*_0 _= *F*(*W*_0 _*a*_0_). Examples of steady-state solutions are plotted for various choices of *L *in Figure 3. (Note that the sudden drop to zero right on the boundaries reflects the nonlocal nature of the neural field equation.) Next, we consider a periodic domain with *w*(*x*) = *w*(*x *+ *L*) for all *x *∈ [0, *L*]. One way to generate such a weight distribution is to take $w\left(x\right)=\sum_{n\in \mathbb{Z}}w_{0}\left(x+nL\right)$ with *w*_0_(*x*) given by the Gaussian distribution of Equation 2.6. In this case, there is a unique nontrivial steady-state solution *a*(*x*) = *a*_0 _for all *x*. For both choices of boundary condition, there still exists the unstable zero-activity state *a*(*x*) *≡ *0. Now, we suppose that some source of multiplicative noise is added to the neural field equation, which vanishes when *a*(*x, t*) = 0. It is then possible that noise-induced fluctuations drive the system to the zero-activity state, resulting in the extinction of all activity since the noise also vanishes there. Assuming that the noise is weak, the time to extinction will be exponentially large, so it is very unlikely to occur during the passage of the invasive front.

In the remainder of the paper, we consider two distinct problems associated with the presence of an unstable zero-activity state in the neural field model (Equation 2.1). First, how do slowly varying spatial heterogeneities in the synaptic weights affect the speed of propagating pulled fronts (Sections 3 and 4)? Second, what is the estimated time for extinction of a stable steady state in the presence of multiplicative extrinsic noise (Sections 5 and 6)? Both problems are linked from a methodological perspective since the corresponding analysis reduces to solving an effective Hamiltonian dynamical system.

## 3 Hamilton-Jacobi dynamics and slow spatial heterogeneities

Most studies of neural fields assume that the synaptic weight distribution only depends upon the distance between interacting populations, that is, *w*(*x, y*) = *w*(|*x *- *y*|). This implies translation symmetry of the underlying integrodifferential equations (in an unbounded or periodic domain). However, if one looks more closely at the anatomy of the cortex, it is clear that it is far from homogeneous, having a structure at multiple spatial scales. For example, to a first approximation, the primary visual cortex (V1) has a periodic-like microstructure on the millimeter length scale, reflecting the existence of various stimulus feature maps. This has motivated a number of studies concerned with the effects of a periodically modulated weight distribution on front propagation in neural fields [41-43,51]. A typical example of a periodically modulated weight distribution is

(3.1) w(x,y)=w(x-y)[1+K(y/ε)],

where 2*πε *is the period of the modulation with *K*(*x*) = *K*(*x *+ 2*π*) for all *x*. In the case of a sigmoidal or Heaviside nonlinearity, two alternative methods for analyzing the effects of periodic wave modulation have been used: one is based on homogenization theory for small *ε *[41,51], and the other is based on analyzing interfacial dynamics [42,43]. Both approaches make use of the observation that for sufficiently small amplitude modulations, numerical simulations of the inhomogeneous network show a front-like wave separating high and low activity metastable states. However, the wave does not propagate with constant speed but oscillates periodically in an appropriately chosen moving frame. This pulsating front solution satisfies the periodicity condition *a*(*x, t*) = *a*(*x *+ *Δ, t *+ *T*), so we can define the mean speed of the wave to be *c *= *Δ/T*.

Recently, Coombes and Laing [42] have analyzed the propagation of pulsating *pulled *fronts in a neural field with periodically modulated weights, extending the previous work of Shigesada et al. on reaction-diffusion models of the spatial spread of invading species into heterogeneous environments [5,12]. We briefly sketch the basic steps in the analysis. First, we substitute the periodically modulated weight distribution (Equation 3.1) into Equation 2.1 and linearize about the leading edge of the wave where *a*(*x, t*) ~ 0:

(3.2) $$\frac{\partial a\left(x,t\right)}{\partial t}=-a\left(x,t\right)+\int_{-\infty }^{\infty }w\left(x-y\right)\left[1+K\left(y/\varepsilon \right)\right]a\left(y,t\right)dy.$$

Now, we assume a solution of the form *a*(*x, t*) = *A*(*ξ*)*P*(*x*), *ξ *= *x *- *ct *with *A*(*ξ*) → 0 as *ξ *→ ∞ and *P*(*x *+ 2*πε*) = *P*(*x*). Substitution into Equation 3.2 then gives

(3.3) $$-cP\left(x\right)A^{\prime }\left(\xi \right)=-P\left(x\right)A\left(\xi \right)+\int_{-\infty }^{\infty }w\left(x-y\right)\left[1+K\left(y/\varepsilon \right)\right]P\left(y\right)A\left(\xi -\left[x-y\right]\right)dy.$$

Taking *A*(*ξ*) ~ e^-*λξ *^and substituting into the above equation yield a nonlocal version of the Hill equation:

(3.4) $$\left(1+c\lambda \right)P\left(x\right)=\int_{-\infty }^{\infty }{\text{e}}^{\lambda \left[x-y\right]}w\left(x-y\right)\left[1+K\left(y/\varepsilon \right)\right]P\left(y\right)dy.$$

**Figure 4:**
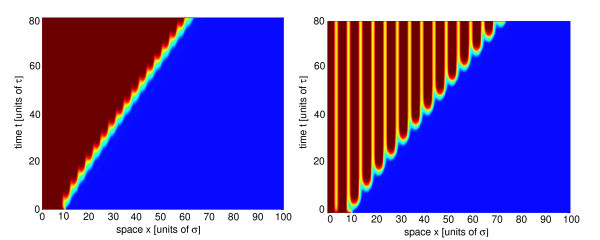
**Space-time contour plots of a pulsating front**. Space-time contour plots of a pulsating front solution of the neural field equation (Equation 3.2) with piecewise linear firing rate function (Equation 2.3), Gaussian weight distribution (Equation 2.6), and a 2*πε*-periodic modulation of the synaptic weights, *K*(*x*) = cos(*x/ε*). (**a**) *ε *= 0.5 and (**b**) *ε *= 0.8. Other parameters are *W*_0 _= 1.2, *σ *= 1.0, and *κ *= 0.4.

In order to determine the minimal wave speed, it is necessary to find a bounded periodic solution *P*(*x*) of Equation 3.4, which yields a corresponding dispersion relation *c *= *c*(*λ*), whose minimum with respect to *λ *can then be determined (assuming it exists). One way to obtain an approximate solution to Equation 3.4 is to use Fourier methods to derive an infinite matrix equation for the Fourier coefficients of the periodic function *P*(*x*), and then to numerically solve a finite truncated version of the matrix equation. This is the approach followed in [42]. The matrix equation takes the form

(3.5) $$\left(1+c\lambda \right)P_{m}=W\left(\lambda -im/\varepsilon \right)P_{m}+W\left(\lambda -im/\varepsilon \right)\underset{l}{\sum }K_{l}P_{m-l},$$

where *K*(*x/ε*) = Σ_*n *_*K*_*n*_e^*imx/ε *^and *P*(*x*) = Σ_*n *_*P*_*n*_e^*imx/ε*^. One finds that the mean velocity of a pulsating front increases with the period 2*πε *of the synaptic modulations [42]. This is illustrated in Figure 4, where we show space-time plots of a pulsating front for *ε *= 0.5 and *ε *= 0.8.

In this section, we develop an alternative method for analyzing pulled fronts in heterogeneous neural fields, based upon the Hamilton-Jacobi dynamics of sharp interfaces, which is particularly applicable to slowly varying spatial modulations [10,16-19]. That is, we consider a heterogeneous version of the neural field equation (Equation 2.1) of the form

(3.6) $$\frac{\partial a\left(x,t\right)}{\partial t}=-a\left(x,t\right)+F\left(\int_{-\infty }^{\infty }w\left(x-x^{\prime }\right)J\left(\varepsilon x^{\prime }\right)a\left(x^{\prime },t\right)dx^{\prime }\right),$$

in which there is a slow (nonperiodic) spatial modulation *J*(*εx*) of the synaptic weight distribution with *ε *≪ 1. The synaptic heterogeneity is assumed to occur on a longer spatial scale than the periodic-like microstructures associated with stimulus feature maps. Although we do not have a specific example of long wavelength modulations in mind, we conjecture that these might be associated with inter-area cortical connections. For example, it has been shown that heterogeneities arise as one approaches the V1/V2 border in the visual cortex, which has a number of effects including the generation of reflected waves [52]. It is not yet clear how sharp is the transition across the V1/V2 border.

The first step in the Hamilton-Jacobi method is to rescale space and time in Equation 3.6 according to *t *→ *t/ε *and *x *→ *x/ε *[10,18,19]:

(3.7) $$\varepsilon \frac{\partial a\left(x,t\right)}{\partial t}=-a\left(x,t\right)+F\left(\frac{1}{\varepsilon }\int_{-\infty }^{\infty }w\;\left(\left[x-x^{\prime }\right]/\varepsilon \right)J\left(x^{\prime }\right)a\left(x^{\prime },t\right)dx^{\prime }\right).$$

Under this hyperbolic rescaling, the front region where the activity *a*(*x, t*) rapidly increases as *x *decreases from infinity becomes a step as *ε *→ 0 (see Figure 2b). This motivates introducing the WKB approximation

(3.8) $$a\left(x,t\right)\sim {\text{e}}^{-G\left(x,t\right)/\varepsilon }$$

with *G*(*x, t*) *>*0 for all *x > x*(*t*) and *G*(*x*(*t*), *t*) = 0. The point *x*(*t*) determines the location of the front and *c *= *ẋ*. Substituting Equation 3.8 into Equation 3.7 gives

(3.9) $$-\partial_{t}G\left(x,t\right)=-1+\frac{1}{\varepsilon }\int_{-\infty }^{\infty }w\left(\left[x-x^{\prime }\right]/\varepsilon \right)J\left(x^{\prime }\right){\text{e}}^{-[G\left(x^{\prime },t\right)-G\left(x,t\right)/\varepsilon }dx^{\prime }.$$

We have used the fact that for *x > x*(*t*) and *ε *≪ 1, the solution is in the leading edge of the front, so we can take *F *to be linear.

In order to simplify Equation 3.9, we use the method of steepest descents. First, we introduce the Fourier transform of the weight distribution *w*(*x*) according to

(3.10) $$w\left(x\right)=\int_{-\infty }^{\infty }\tilde{w}\left(k\right){\text{e}}^{ikx}\frac{dk}{2\pi }.$$

Substituting into Equation 3.9 and reversing the order of integration give

(3.11) $$-\partial_{t}G\left(x,t\right)=-1+\frac{1}{\varepsilon }\int_{-\infty }^{\infty }\int_{-\infty }^{\infty }\tilde{w}\left(k\right)J\left(x^{\prime }\right){\text{e}}^{-S\left(k,x^{\prime };x,t\right)/\varepsilon }dx^{\prime }\frac{dk}{2\pi },$$

where

(3.12) $$S\left(k,x^{\prime };x,t\right)=ik\left(x^{\prime }-x\right)+G\left(x^{\prime },t\right)-G\left(x,t\right).$$

Exploiting the fact that *ε *is small, we perform steepest descents with respect to the *x' *variable with (*k, x, t*) fixed. Let *x' *= *z*(*k, t*) denote the stationary point for which *∂S/∂x' *= 0, which is given by the solution to the implicit equation

(3.13) $$ik+\partial_{x}G\left(x^{\prime },t\right)=0.$$

Taylor expanding *S *about this point (assuming it is unique) gives to second order

(3.14) $$\begin{array}{ccc} S\left(k,x^{\prime };x,t\right) & \approx S\left(k,z\left(k,t\right);x,t\right)+{\left(\frac{1}{2}\frac{\partial^{2}S}{\partial {x^{\prime }}^{2}}\right|}_{x^{\prime }=z\left(k,t\right)}{\left(x^{\prime }-z\left(k,t\right)\right)}^{2} & \\ & =ik\left[z\left(k,t\right)-x\right]+G\left(z\left(k,t\right),t\right)-G\left(x,t\right)\\ & \;-\frac{1}{2}\partial_{xx}G\left(z\left(k,t\right),t\right){\left(x^{\prime }-z\left(k,t\right)\right)}^{2}. \end{array}$$

Substituting into Equation 3.11 and performing the resulting Gaussian integral with respect to *x*' yield the result

(3.15) $$\begin{array}{ccc} -\partial_{t}G\left(x,t\right)= & -1+\frac{1}{\varepsilon }\int_{-\infty }^{\infty }\sqrt{\frac{2\pi \varepsilon }{\partial_{xx}G\left(z\left(k,t\right),t\right)}}\tilde{w}\left(k\right)J\left(z\left(k,t\right)\right) & \\ & \;\times {\text{e}}^{-\left(ik\left[z\left(k,t\right)-x\right]+G\left(z\left(k,t\right),t\right)-G\left(x,t\right)\right)/\varepsilon }\frac{dk}{2\pi }. \end{array}$$

This can be rewritten in the form

(3.16) $$-\partial_{t}G\left(x,t\right)=-1+\frac{1}{\sqrt{2\pi \varepsilon }}\int_{-\infty }^{\infty }\tilde{w}\left(k\right)J\left(z\left(k,t\right)\right){\text{e}}^{-\hat{S}\left(k;x,t\right)/\varepsilon }dk,$$

where

(3.17) $$\hat{S}\left(k;x,t\right)=ik\left[z\left(k,t\right)-x\right]+G\left(z\left(k,t\right)\;t\right)-G\left(x,t\right)+\frac{\varepsilon }{2}\text{ln}\;\partial_{xx}G\left(z\left(k,t\right),t\right).\;$$

The integral over *k *can also be evaluated using steepest descents. Thus, we Taylor expand *Ŝ *to second order about the stationary point *k *= *k*(*x, t*), which is the solution to the equation

(3.18) $$0=\frac{\partial \hat{S}}{\partial k}=i\left[z\left(k,t\right)-x\right]+\frac{\partial z\left(k,t\right)}{\partial k}\left[ik+\partial_{x}G\left(z\left(k,t\right),t\right)+\frac{\varepsilon }{2}\frac{\partial_{xxx}G\left(z\left(k,t\right),t\right)}{\partial_{xxx}G\left(z\left(k,t\right),t\right)}\right].$$

It follows from Equations 3.13 and 3.18 that $z\left(k\left(x,t\right),t\right)=x+O\left(\varepsilon \right)$, so

(3.19) $$k\left(x,t\right)=i\partial_{x}G\left(x,t\right)+O\left(\varepsilon \right).$$

Moreover,

(3.20) $$\hat{S}\left(k;x,t\right)\approx {\left(\frac{1}{2}\frac{\partial^{2}\hat{S}}{\partial k^{2}}\right|}_{k=k\left(x,t\right)}{\left(k-k\left(x,t\right)\right)}^{2}.$$

Substituting into Equation 3.16 and performing the Gaussian integral with respect to *k *give to leading order in *ε*

(3.21) $$-\partial_{t}G\left(x,t\right)=-1+\frac{1}{\sqrt{i\partial_{xx}G\left(x,t\right)\partial_{k}z\left(k\left(x,t\right)t\right)}}\tilde{w}\left(k\left(x,t\right)\right)J\left(x\right).$$

Finally, setting *x*' = *z*(*k, t*) in Equation 3.13 and differentiating with respect to *k *show that *∂_xx_G*(*z*(*k, t*), *t*)*∂_k_z*(*k, t*) = -*i*; hence,

(3.22) $$-\partial_{t}G\left(x,t\right)=-1+\tilde{w}\left(i\partial_{x}G\left(x,t\right)\right)J\left(x\right).$$

Equation 3.22 is formally equivalent to the Hamilton-Jacobi equation

(3.23) $$\partial_{t}G+H\left(\partial_{x}G,x\right)=0$$

with corresponding Hamiltonian

(3.24) $$H\left(p,x\right)=-1+\tilde{w}\left(ip\right)J\left(x\right),$$

where *p *= *∂_x_G *is interpreted as the conjugate momentum of *x*, and

(3.25) $$\tilde{w}\left(ip\right)=\hat{W}\left(p\right)+\hat{W}\left(-p\right)\equiv W\left(p\right)$$

with $\hat{W}\left(p\right)$ as the Laplace transform of *w*(*x*). It follows that the Hamilton-Jacobi equation (Equation 3.23) can be solved in terms of the Hamilton equations

(3.26) $$\frac{dx}{ds}=\frac{\partial H}{\partial p}=J\left(x\right)W^{\prime }\left(p\right)=J\left(x\right)\left[\hat{W^{\prime }}\left(p\right)-\hat{W^{\prime }}\left(-p\right)\right]$$

(3.27) $$\frac{dp}{ds}=-\frac{\partial H}{\partial x}=-J^{\prime }\left(x\right)W\left(p\right).$$

Let *X*(*s*; *x, t*) and *P *(*s*; *x, t*) denote the solution with *x*(0) = 0 and *x*(*t*) = *x*. We can then determine *G*(*x, t*) according to

(3.28) $$G\left(x,t\right)=-E\left(x,t\right)t+\int_{0}^{t}P\left(s;x,t\right)\dot{X}\left(s;x,t\right)ds.$$

Here,

(3.29) E(x,t)=H(P(s;x,t),X(s;x,t)),

which is independent of *s *due to conservation of 'energy,' that is, the Hamiltonian is not an explicit function of time.

## 4 Calculation of wave speed

### 4.1 Homogeneous case: *J*(*x*) *≡ *1

Let us begin by rederiving the wave speed for a homogeneous neural field (see Section 2) by setting *J*(*x*) *≡ *1. In this case, *dp/ds *= 0, so *p *= *λ*_0 _independently of *s*. Hence, *x*(*s*) = *xs/t*, which implies that

(4.1) $$W^{\prime }\left(\lambda_{0}\right)=\frac{x}{t}.$$

By construction, the location *x*(*t*) of the front at time *t *is determined by the equation *G*(*x*(*t*), *t*) = 0. Differentiating with respect to *t *shows that $\dot{x}\partial_{x}G+\partial_{t}G=0$ or

(4.2) $$\dot{x}=-\frac{\partial_{t}G}{\partial_{x}G}=\frac{-1+W\left(\lambda_{0}\right)}{\lambda_{0}}.$$

It follows that *λ*_0 _is given by the minimum of the function

(4.3) $$c\left(\lambda \right)=\frac{-1+W\left(\lambda \right)}{\lambda }$$

and *c*_0 _= *c*(*λ*_0_). This recovers the result of Section 2. Thus, in the case of a Gaussian weight distribution, *λ*_0 _is related to *c*_0 _according to Equation 2.15.

### 4.2 Synaptic heterogeneity: *J*(*x*) = 1 + *β f*(*x*), 0 *β *≪ 1

Suppose that there exists a small amplitude, slow modulation of the synaptic weights *J*(*x*) = 1 + *βf*(*x*) with *β *≪ 1. We can then obtain an approximate solution of Hamilton's equations (Equations 3.26 and 3.27) and the corresponding wave speed using regular perturbation theory along analogous lines to a previous study of the F-KPP equation [10]. We introduce the perturbation expansions

(4.4) $$x\left(s\right)=x_{0}\left(s\right)+\beta x_{1}\left(s\right)+O\left(\beta^{2}\right),\;p\left(s\right)=p_{0}\left(s\right)+\beta p_{1}\left(s\right)+O\left(\beta^{2}\right)$$

and substitute into Equations 3.26 and 3.27. Taylor expanding the nonlinear function *f*(*x*) about *x*_0 _and $W\left(p\right)=\hat{W}\left(p\right)+\hat{W}\left(-p\right)$ about *p*_0 _then leads to a hierarchy of equations, the first two of which are

(4.5) $${\dot{p}}_{0}\left(s\right)=0,\;{\dot{x}}_{0}\left(s\right)=W^{\prime }\left(p_{0}\right),$$

and

(4.6) $${\dot{p}}_{1}\left(s\right)=-f^{\prime }\left(x_{0}\right)W\left(p_{0}\right),\;{\dot{x}}_{1}\left(s\right)=W^{\prime \prime }\left(p_{0}\right)p_{1}\left(s\right)+f\left(x_{0}\right)W^{\prime }\left(p_{0}\right).$$

These are supplemented by the Cauchy conditions *x*_0_(0) = 0, *x*_0_(*t*) = *x*, and *x_n_*(0) = *x_n_*(*t*) = 0 for all integers *n ≥ *1. Equations 4.5 have solutions of the form

(4.7) $$p_{0}\left(s\right)=\lambda ,\;x_{0}\left(s\right)=W^{\prime }\left(\lambda \right)s+B_{0}$$

with *λ *and *B*_0 _independent of *s*. Imposing the Cauchy data then implies that *B*_0 _= 0 and *λ *satisfy the equation

(4.8) $$W^{\prime }\left(\lambda \right)=x/)t.$$

At the next order, we have the solutions

(4.9) $$p_{1}\left(s\right)=-W\left(\lambda \right)\frac{t}{x}f\left(xs/t\right)+A_{1},$$

(4.10) $$\begin{array}{ccc} x_{1}\left(s\right)= & -W^{\prime \prime }\left(\lambda \right)W\left(\lambda \right)\frac{t^{2}}{x^{2}}\int_{0}^{xs/t}f\left(y\right)dy & \\ & \;+\int_{0}^{xs/t}f\left(y\right)dy+W^{\prime \prime }\left(\lambda \right)A_{1}s+B_{1}, \end{array}$$

with *A*_1 _and *B*_1 _independent of *s*. Imposing the Cauchy data then implies that *B*_1 _= 0 and

(4.11) $$A_{1}=A_{1}\left(x,t\right)=W\left(\lambda \right)\frac{t}{x^{2}}\int_{0}^{x}f\left(y\right)dy-\frac{1}{tW^{\prime \prime }\left(\lambda \right)}\int_{0}^{x}f\left(y\right)dy.$$

Given these solutions, the energy function *E*(*x, t*) is

(4.12) $$\begin{array}{ccc} E\left(x,t\right) & =-1+\left[1+\beta f\left(x_{0}+\beta x_{1}+\ldots \right)\right]W\left(\lambda +\beta p_{1}+\ldots \right) & \\ & =-1+W\left(\lambda \right)+\beta \left[W^{\prime }\left(\lambda \right)p_{1}\left(s\right)+f\left(x_{0}\left(s\right)\right)W\left(\lambda \right)\right]+O\left(\beta^{2}\right). \end{array}$$

Substituting for *x*_0_(*s*) and *p*_1_(*s*) and using the condition $W^{\prime }\left(\lambda \right)=x/)t$, we find that

(4.13) $$E\left(x,t\right)=-1+W\left(\lambda \right)+\beta \frac{x}{t}A_{1}\left(x,t\right)+O\left(\beta^{2}\right),$$

which is independent of *s *as expected. Similarly,

(4.14) $$\begin{array}{ccc} \int_{0}^{t}p\left(s\right)\dot{x}\;\left(s\right)ds & =\lambda x+\beta W^{\prime }\left(\lambda \right)\int_{0}^{t}p_{1}\left(s\right)ds+O\left(\beta^{2}\right) & \\ & =\lambda x+\beta \frac{W^{\prime }\left(\lambda \right)}{W^{\prime \prime }\left(\lambda \right)}\int_{0}^{t}\left[{\dot{x}}_{1}\left(s\right)-W^{\prime }\left(\lambda \right)fW^{\prime }\left(\lambda \right)s\right]ds+O\left(\beta^{2}\right)\\ & =\lambda x-\beta \frac{W^{\prime }\left(\lambda \right)}{W^{\prime \prime }\left(\lambda \right)}\int_{0}^{x}f\left(y\right)dy+O\left(\beta^{2}\right). \end{array}$$

Hence, to first order in *β*,

(4.15) $$G\left(x,t\right)=t-W\left(\lambda \right)t+\lambda x-\beta W\left(\lambda \right)\frac{t}{x}\int_{0}^{x}f\left(y\right)dy.$$

We can now determine the wave speed *c *by imposing the condition *G*(*x*(*t*), *t*) = 0 and performing the perturbation expansions $x\left(t\right)=x_{0}\left(t\right)\beta x_{1}\left(t\right)+O\left(\beta^{2}\right)$ and $\lambda =\lambda_{0}+\beta \lambda_{1}+O\left(\beta^{2}\right)$. Substituting into Equation 4.15 and collecting terms at $O\left(1\right)$ and $O\left(\beta \right)$ lead to the following result:

(4.16) $$x\left(t\right)=c_{0}t+\frac{\beta W\left(\lambda_{0}\right)}{c_{0}\lambda_{0}}\int_{0}^{c_{0}t}f\left(y\right)dy+O\left(\beta^{2}\right).$$

Here, *c*_0 _is the wave speed of the homogeneous neural field (*β *= 0), which is given by *c*_0 _= *c*(*λ*_0_) with *λ*_0 _obtained by minimizing the function *c*(*λ*) defined by Equation 4.3 (see Equation 2.15). Finally, differentiating both sides with respect to *t *and inverting the hyperbolic scaling yield

(4.17) $$c\equiv \dot{x}\left(t\right)=c_{0}+\frac{\beta W\left(\lambda_{0}\right)}{\lambda_{0}}f\left(\varepsilon c_{0}t\right)+O\left(\beta^{2}\right).$$

### 4.3 Numerical results

**Figure 5:**
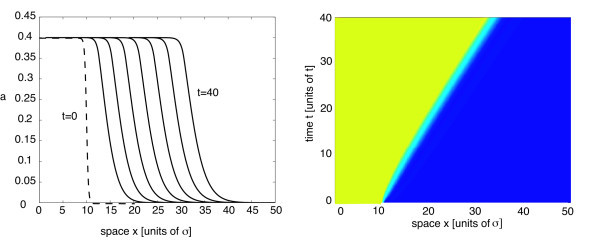
**Propagating front in a homogeneous network**. Propagating front in a homogeneous network with Gaussian weights (Equation 2.6) and piecewise linear rate function (Equation 2.3). Parameter values are *W*_0 _= 1.2, *σ *= 1, and *κ *= 0.4. (**a**) Snapshots of wave profile at time intervals of width *Δt *= 5 from *t *= 10 to *t *= 40. (**b**) Space-time contour plot. Wave speed asymptotically approaches the minimum *c*_0 _of the velocity dispersion curve given by Equation 2.12.

**Figure 6:**
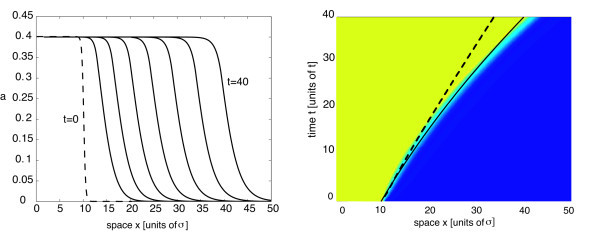
**Propagating front in a network with a linear heterogeneity (*ε*^2 ^= 0.005)**. Propagating front in a network with a linear heterogeneity in the synaptic weights, *J*(*x*) = 1 + *ε*(*x - l*), *l *= 10, and *ε*^2 ^= 0.005. Other parameters as in Figure 5. (**a**) Snapshots of wave profile at time intervals of width *Δt *= 5 from *t *= 10 to *t *= 40. (**b**) Space-time contour plot. Wave speed increases approximately linearly with time, so the position *x*(*t*) of the front evolves according to a downward parabola. Theoretical curve based on the perturbation calculation is shown by the solid curve. The trajectory of the front in the corresponding homogeneous case (see Figure 5b) is indicated by the dashed curve.

**Figure 7:**
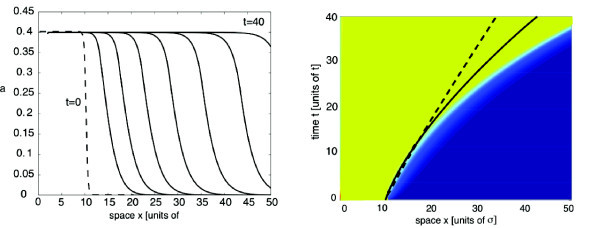
**Propagating front in a network with a linear heterogeneity (*ε*^2 ^= 0.01)**. Propagating front in a network with a linear heterogeneity in the synaptic weights, *J*(*x*) = 1+*ε*(*x*-*l*), *l *= 10, and *ε*^2 ^= 0.01. Other parameters as in Figure 5. (**a**) Snapshots of wave profile at time intervals of width *Δt *= 5 from *t *= 10 to *t *= 40. (**b**) Space-time contour plot. Wave speed increases approximately linearly with time, so the position *x*(*t*) of the front evolves according to a downward parabola. Theoretical curve based on the perturbation calculation is shown by the solid curve. The trajectory of the front in the corresponding homogeneous case (see Figure 5b) is indicated by the dashed curve.

In order to compare our analytical results with computer simulations, we numerically solve a discretized version of the neural field equation (Equation 3.6) using a direct Euler scheme. We take -*L ≤ × ≤ L *with free boundary conditions and an initial condition given by a steep sigmoid

(4.18) $$a\left(x,0\right)=\frac{0.5}{1+\text{exp}\left(-\eta \left(x-l\right)\right)},$$

with *η *= 5, where *l *determines the approximate initial position of the front. For concreteness, *L *= 100 and *l *= 10. Space and time units are fixed by setting the range of synaptic weights *σ *= 1 and the time constant *τ *= 1. In Figure 5a, we show snapshots of a pulled front in the case of a homogeneous network with Gaussian weights (Equation 2.6) and piecewise linear firing rate function (Equation 2.3). A corresponding space-time plot is given in Figure 5b, which illustrates that the speed of the front asymptotically approaches the calculated minimal wave speed *c*_0_. (Note that pulled fronts take an extremely long time to approach the minimal wave speed at high levels of numerical accuracy since the asymptotics are algebraic rather than exponential in time [53].) In Figures 6 and 7, we plot the corresponding results in the case of an inhomogeneous network. For the sake of illustration, we choose the synaptic heterogeneity to be a linear function of displacement, that is, *J*(*x*) = 1 + *ε*(*x - l*), where for the sake of illustration, we have set *β *= *ε*. Equation 4.16 implies that

(4.19) $$\begin{array}{ccc} x\left(t\right) & =l+c_{0}t+\frac{\varepsilon^{2}W\left(\lambda_{0}\right)}{2c_{0}\lambda_{0}}\left[{\left(c_{0}t\right)}^{2}-2c_{0}lt\right] & \\ & =l+\left[c_{0}-\frac{\varepsilon^{2}l\left(c_{0}\lambda_{0}+1\right)}{\lambda_{0}}\right]t+\frac{\varepsilon^{2}c_{0}\left(c_{0}\lambda_{0}+1\right)}{2\lambda_{0}}t^{2}, \end{array}$$

where we have used Equation 4.3. We assume that the initial position of the front is *x*(0) = *l*. Hence, our perturbation theory predicts that a linearly increasing modulation in synaptic weights results in the leading edge of the front tracing out a downward parabola in a space-time plot for times $t\ll O\left(1/\varepsilon^{2}\right)$. This is consistent with our numerical simulations for *ε*^2 ^= 0.005, as can be seen in the space-time plot of Figure 6b. However, our approximation for the time-dependent speed breaks down when $t=O\left(1/\varepsilon^{2}\right)$, as illustrated in Figure 7c for *ε*^2 ^= 0.01.

## 5 Path integral formulation of a stochastic neural field

We now turn to the second main topic of this paper, namely noise-induced transitions to extinction in the presence of a zero absorbing state. Therefore, let us modify the scalar neural field equation (Equation 2.1) by adding extrinsic multiplicative noise. The resulting Langevin equation is

(5.1) $$dA=\left[-A+F\left(\int_{0}^{L}w\left(x-y\right)A\left(y,t\right)dy\right)\right]dt+\varepsilon g\left(A\right)dW\left(x,t\right),$$

for 0 *≤ t ≤ T *and initial condition *A*(*x*, 0) = *Φ*(*x*). We take *dW *(*x, t*) to represent an independent Wiener process such that

(5.2) $$\left\langle dW\left(x,t\right)\right\rangle =0,\;\left\langle dW\left(x,t\right)dW\left(x^{\prime },t^{\prime }\right)\right\rangle =2LC\left(\left[x-x^{\prime }\right]/)\lambda \right)\delta \left(t-t^{\prime }\right)dtdt^{\prime }.$$

Here, *λ *is the spatial correlation length of the noise such that *C*(*x/λ*) → *δ*(*x*) in the limit *λ *→ 0, and *ε *determines the strength of the noise, which is assumed to be weak. We will assume that *g*(0) = 0, so the zero-activity state *A *= 0 is an absorbing state of the system; any noise-induced transition to this state would then result in the extinction of all activity. An example of multiplicative noise that vanishes at *A *= 0 is obtained by carrying out a diffusion approximation of the neural master equation previously introduced by Buice et al. [46,54] (see Bressloff [55,56]). Before considering the problem of extinction, a more immediate question is how multiplicative noise affects the propagation of the invasive pulled fronts analyzed in Section 2. Since this particular issue is addressed in some detail elsewhere [49], we only summarize the main findings here.

In the case of the F-KPP equation with multiplicative noise, it has previously been shown that the stochastic wandering of a pulled front about its mean position is subdiffusive with var*Δ*(*t*) ~ *t*^1/2^, in contrast to the diffusive wandering of a front propagating into a metastable state for which var*Δ*(*t*) ~ *t *[11]. Such scaling is a consequence of the asymptotic relaxation of the leading edge of the deterministic pulled front. Since pulled front solutions of the neural field equation (Equation 2.1) exhibit similar dynamics, it suggests that there will also be subdiffusive wandering of these fronts in the presence of multiplicative noise. This is indeed found to be the case [49]. More specifically, the multiplicative noise term in Equation 5.1 generates two distinct phenomena that occur on different time scales: a diffusive-like displacement of the front from its uniformly translating position at long time scales, and fluctuations in the front profile around its instantaneous position at short time scales. This can be captured by expressing the solution *A *of Equation 5.1 as a combination of a fixed wave profile *A*_0 _that is displaced by an amount *Δ*(*t*) from its uniformly translating mean position *ξ *= *x - ct*, and a time-dependent fluctuation *Φ *in the front shape about the instantaneous position of the front:

(5.3) $$A\left(x,t\right)=A_{0}\left(\xi -\Delta \left(t\right)\right)+\varepsilon \Phi \left(\xi -\Delta \left(t\right),t\right).$$

**Figure 8:**
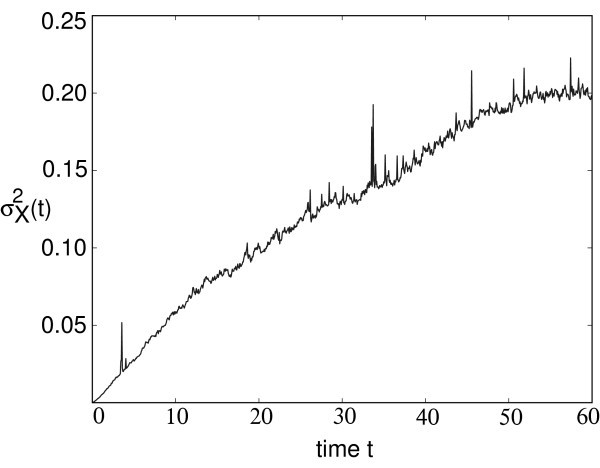
**Plot of variance in position of a stochastic pulled front**. Plot of variance $\sigma_{X}^{2}\left(t\right)$ of the position of a stochastic pulled front as a function of time for noise amplitude *ε *= 0.005 and $g\left(A\right)=A/)\sqrt{L}$. Other parameters are *W*_0 _= 1.2, *κ *= 0.8, and *σ *= 1.

Here, *c *denotes the mean speed of the front. (In the Stratonovich version of multiplicative noise, there is an *ε*-dependent shift in the speed *c*.) Numerical simulations of Equation 5.1 with *F *given by the piecewise linear firing rate (Equation 2.3) and *g*(*A*) = *A *are consistent with subdiffusive wandering of the front, as illustrated in Figure 8. The variance $\sigma_{X}^{2}\left(t\right)$ is obtained by averaging over level sets [49]. That is, we determine the positions *X_z_*(*t*) such that *A*(*X_z_*(*t*), *t*) = *z *for various level set values *z *∈ (0.5*κ*, 1.3*κ*) and then define the mean location to be $\bar{X}\left(t\right)=E\left[X_{z}\left(t\right)\right]$, where the expectation is first taken with respect to the sampled values *z *and then averaged over *N *trials. The corresponding variance is given by $\sigma_{X}^{2}\left(t\right)=E\left[{\left(X_{z}\left(t\right)-\bar{X}\left(t\right)\right)}^{2}\right]$. It can be seen that the variance exhibits subdiffusive behavior over long time scales.

In order to develop a framework to study rare extinction events in the weak noise limit, we construct a path integral representation of the stochastic Langevin equation (Equation 5.1). We will assume that the multiplicative noise is of Ito form [57]. For reviews on path integral methods for stochastic differential equations, see [58-60]. Discretizing both space and time with *A_i, m _*= *A*(*mΔd, iΔt*), *W_i, m _*= *Δd*^-1/2^*W*(*mΔd, iΔt*), and *Δdw_mn _*= *w*(*mΔd, nΔd*) gives

$$\begin{array}{ccc} A_{i+1,m}-A_{i,m} & =\left[A_{i,m}+F\left(\Delta d\underset{n}{\sum }w_{mn}A_{i,n}\right)\right]\Delta t & \\ & \;+\varepsilon \frac{\sqrt{\Delta t}}{\sqrt{\Delta d}}g\left(A_{i,m}\right)dW_{i,m}, \end{array}$$

with *i *= 0, 1, ..., *N, T *= *NΔt*, and

(5.4) $$\left\langle dW_{i,m}\right\rangle =0,\;\left\langle dW_{i,m}dW_{i^{\prime },m^{\prime }}\right\rangle =L\delta_{i,i^{\prime }}\delta_{m,m^{\prime }}.$$

Let **A **and **W **denote the vectors with components *A_i, m _*and *W_i, m_*, respectively. Formally, the conditional probability density function for **A **given a particular realization of the stochastic process **W **and initial condition *A*_0, *m *_= *Φ_m _*is

(5.5) $$\begin{array}{ccc} P[A\text{|}W\text{]} & =\prod_{n}\prod_{i=0}^{N}\delta (A_{i+1,m}-A_{i,m}+\left[A_{i,m}-F\left(\Delta d\sum_{n}w_{mn}A_{i,n}\right)\right]\;\Delta t & \\ & \;\;\;\;\;\;\;\;-\varepsilon \frac{\sqrt{\Delta t}}{\sqrt{\Delta d}}g\left(A_{i,m}\right)dW_{i,m}). \end{array}$$

Inserting the Fourier representation of the Dirac delta function,

(5.6) $$\delta \left(A_{i,m}\right)=\frac{1}{2\pi }\int {\text{e}}^{-i{\tilde{A}}_{i,m}\;A_{i,m}}d{\tilde{A}}_{i,m},$$

gives

(5.7) $$\begin{array}{ccc} P[A\text{|}W\text{]} & =\int \prod_{n}\prod_{j=0}^{N}\frac{d{\tilde{A}}_{j,n}}{2\pi }\text{exp}\left\{-i\sum_{i,m}{\tilde{A}}_{i,m}\left(A_{i+1,m}-A_{i,m}\right)\right\} & \\ & \;\times \text{exp}\left\{-i\sum_{i,m}{\tilde{A}}_{i,m}\left[A_{i,m}-F\left(\Delta d\sum_{n}w_{mn}A_{i,n}\right)\right]\Delta t\right\}\\ & \;\;\times \text{exp}\left\{i\sum_{i,m}{\tilde{A}}_{i,m}\left(\varepsilon \frac{\sqrt{\Delta t}}{\sqrt{\Delta d}}g\left(A_{i,m}\right)dW_{i,m}\right)\right\}. \end{array}$$

For a Gaussian white noise process, *W_i, n _*has the probability density function $P\left(W_{i,m}\right)={\left(2\pi L\right)}^{-1/2}e^{-W_{i,m}^{2}/2L}$. Hence, setting

$$P[A\text{] = }\int P\text{[}A\text{|}W\text{]}\underset{j,n}{\prod }P(W_{j,n})dW_{j,n}$$

and performing the integration with respect to *W_j, n _*by completing the square, we obtain the result

(5.8) $$\begin{array}{ccc} P[A\text{]} & =\int \prod_{n}\prod_{j=0}^{N}\frac{d{\tilde{A}}_{j,n}}{2\pi }\text{exp}\left\{-i\sum_{i,m}{\tilde{A}}_{i,m}\left(A_{i+1,m}-A_{i,m}\right)\right\} & \\ & \;\;\;\;\times \text{exp}\left\{-i\sum_{i,m}{\tilde{A}}_{i,m}\left[A_{i,m}-F\left(\Delta d\sum_{n}w_{mn}A_{i,n}\right)\right]\Delta t\right\}\\ & \;\;\;\;\;\times \text{exp}\left\{\varepsilon^{2}L{\sum_{i,m}\left(i{\tilde{A}}_{i,m}\right)}^{2}g^{2}\left(A_{i,m}\right)\frac{\Delta t}{2\Delta d}\right\}. \end{array}$$

Finally, taking the continuum limits *Δd → *0 and *Δt → *0, *N → ∞ *for fixed *T *with *A_i, m _→ A*(*x, t*) and $i,{\tilde{A}}_{i,m}/\Delta d\to \tilde{A}\left(x,t\right)$ gives the following path integral representation of a stochastic neural field:

(5.9) $$P[A\text{] = }\int D\tilde{A}e^{-S\left[A,\tilde{A}\right]}$$

with

(5.10) $$\begin{array}{ccc} S\left[A,\tilde{A}\right] & =\int_{0}^{L}dx\int_{0}^{T}dt\tilde{A}\left(x,t\right)[At\left(x,t\right)+A\left(x,t\right)-F\left(\int w\left(x-y\right)A\left(y,t\right)dy\right) & \\ & \;\;\;\;\;\;\;\;\;-\frac{\varepsilon^{2}L}{2}\tilde{A}\left(x,t\right)g^{2}\left(A\left(x,t\right)\right)]. \end{array}$$

Given the probability functional *P*[*A*], we can write down path integral representations of various moments of the stochastic field *A*. For example, the mean field is

(5.11) $$\left\langle \left\langle A\left(x,t\right)\right\rangle \right\rangle =\int DAD\tilde{A}A\left(x,t\right){\text{e}}^{-S\left[A,\tilde{A}\right]},$$

whereas the two-point correlation is

(5.12) $$\left\langle \left\langle A\left(x,t\right)A\left(x^{\prime },t^{\prime }\right)\right\rangle \right\rangle =\int D\left[A\right]D\tilde{A}A\left(x,t\right)A\left(x^{\prime },t^{\prime }\right){\text{e}}^{-S\left[A,Ã\right]}.$$

In particular, introducing the conditional probability *p*[*A*_1_, *t|A*_0_, 0] for the initial state *A*(*x*, 0) = *A*_0_(*x*) to be in the final state *A*(*x, t*) = *A*_1_(*x*) at time *t*, we have

(5.13) $${\left(p\text{[}A_{1},t|A_{0},0,],\equiv ,\left\langle \left\langle \underset{x}{\prod }\delta \left(A_{1}\left(x\right)-A\left(x,t\right)\right)\right\rangle \right\rangle ,\int ,D,A,D,\tilde{A},\;,{\text{e}}^{-S\left[A,\tilde{A}\right]}\right|}_{A\left(0\right)=A_{0}}^{A\left(t\right)=A_{1}}.$$

## 6 Hamiltonian-Jacobi dynamics and population extinction in the weak-noise limit

We now use the path integral representation of the conditional probability (Equation 5.13) in order to estimate the time to extinction of a metastable nontrivial state. We proceed along analogous lines to our previous study of a neural master equation with *x*-independent steady states [55,56], where the effective Hamiltonian dynamical system obtained by extremizing the associated path integral action can be used to determine the most probable or optimal path to the zero absorbing state. Alternatively, one could consider a WKB approximation of solutions to the corresponding functional Fokker-Planck equation or master equation, as previously applied to reaction-diffusion systems [21,24-27]. The connection between the two approaches is discussed in [21].

The first step is to perform the rescaling $\tilde{A}\to \tilde{A}/L\varepsilon^{2}$ so that Equation 5.13 becomes

(6.1) $${\left(p\text{[}A_{1},t|A_{0},0,],=,\int ,D,A,D,\tilde{A},\;,{\text{e}}^{-S\left[A,\tilde{A}\right]/)L\varepsilon^{2}}\right|}_{A\left(0\right)=A_{0}}^{A\left(t\right)=A_{1}}.$$

In the limit *ε → *0, the path integral is dominated by the 'classical' solutions *q*(*x, t*), *p*(*x, t*), which extremize the exponent or action of the generating functional:

(6.2) $${\left(\frac{\delta S\left[A,\tilde{A}\right]}{\delta A\left(x,t\right)}\right|}_{\tilde{A}=p,A=q}=0,\;{\left(\frac{\delta S\left[A,\tilde{A}\right]}{\delta \tilde{A}\left(x,t\right)}\right|}_{\tilde{A}=p,A=q}=0$$

These equations reduce to the form

(6.3) $$\frac{\partial q\left(x,t\right)}{\partial t}=\frac{\delta H\left(q,p\right)}{\delta p\left(x,t\right)},\;\frac{\partial p\left(x,t\right)}{\partial t}=\frac{\delta H\left(q,p\right)}{\delta q\left(x,t\right)},$$

where we have set

$$S[q,p\text{] = }\int_{0}^{T}dt\left[\int dx\;p\left(x,t\right)\dot{q}\left(x,t\right)-H[q,p\text{]}\right],$$

such that

(6.4) $$\begin{array}{ccc} H\left[q,p\right] & =\int dx\;p\left(x,t\right)\;[-q\left(x,t\right)+F\left(\int w\left(x-y\right)q\left(y,t\right)dy\right) & \\ & \;+\frac{1}{2}p\left(x,t\right)g^{2}\left(q\left(x,t\right)\right)] \end{array}$$

Equations 6.3 take the form of a Hamiltonian dynamical system in which *q *is a 'coordinate' variable, *p *is its 'conjugate momentum' and ℋ is the Hamiltonian functional. Substituting for ℋ leads to the explicit Hamilton equations:

(6.5) $$\frac{\partial q\left(x,t\right)}{\partial t}\text{ = }-q\left(x,t\right)+F\left(\int w\left(x-y\right)q\left(y,t\right)dy\right)+p\left(x,t\right)g^{2}\left(q\left(x,t\right)\right)$$

(6.6) $$\begin{array}{ccc} \frac{\partial p\left(x,t\right)}{\partial t} & =-p\left(x,t\right)+\int F^{\prime }\left(\int w\left(y-z\right)q\left(z,t\right)dz\right)w\left(y-x\right)p\left(y,t\right)dy & \\ & \;+p^{2}\left(x,t\right)g\left(q\left(x,t\right)\right)g^{\prime }\left(q\left(x,t\right)\right) \end{array}$$

It can be shown that *q*(*x, t*) and *p*(*x, t*) satisfy the same boundary conditions as the physical neural field *A*(*x, t*) [20]. Thus, in the case of periodic boundary conditions, *q*(*x*+*L, t*) = *q*(*x, t*) and *p*(*x*+*L, t*) = *p*(*x, t*). It also follows from the Hamiltonian structure of Equations 6.5 and 6.6 that there is an integral of motion given by the conserved 'energy' *E *= ℋ[*q, p*].

The particular form of ℋ implies that one type of solution is the zero-energy solution *p*(*x, t*) *≡ *0, which implies that *q*(*x, t*) satisfies the deterministic scalar neural field equation (Equation 2.1). In the *t *→ *∞ *limit, the resulting trajectory in the infinite-dimensional phase space converges to the steady-state solution $Q_{+}=\left[q_{s}\left(x\right),0\right]$, where *q_s_*(*x*) satisfies Equation 2.16. The Hamiltonian formulation of extinction events then implies that the most probable path from [*q_s_*(*x*), 0] to the absorbing state is the unique zero-energy trajectory that starts at $Q_{+}$ at time *t *= -∞ and approaches another fixed point $P=\left[0,p_{e}\left(x\right)\right]$ at *t *= +*∞ *[20,21]. In other words, this so-called activation trajectory is a heteroclinic connection $Q_{+}P$ (or instanton solution) in the functional phase space [*q*(*x*), *p*(*x*)]. It can be seen from Equation 6.6 that the activation trajectory is given by the curve

(6.7) $$p\left(x\right)=F_{x}\left[q\right]\equiv \frac{-q\left(x\right)+F\left(\int_{0}^{L}w\left(x-y\right)q\left(y\right)dy\right)}{g{\left(q\left(x\right)\right)}^{2}}$$

so that

(6.8) $$p_{e}\left(x\right)=\underset{q\to 0}{\text{lim}}F_{x}\left[q\right].$$

Note that the condition that *p_e_*(*x*) exists and is finite is equivalent to the condition that there exists a stationary solution to the underlying functional Fokker-Planck equation - this puts restrictions on the allowed form for *g*. For the zero-energy trajectory emanating from $Q_{+}$ at *t *= -*∞*, the corresponding action is given by

(6.9) $$S_{0}=\int_{-\infty }^{\infty }dt\int_{0}^{L}dx\;p\left(x,t\right)\dot{q}\left(x,t\right),$$

and up to pre-exponential factors, the estimated time *τ_e _*to extinction from the steady-state solution *q_s_*(*x*) is given by [20,21]

(6.10) $$\text{ln}\tau_{e}\approx \varepsilon^{-2}\frac{S_{0}}{L}.$$

For *x*-dependent steady-state solutions *q_s_*(*x*), which occur for the Dirichlet boundary conditions and finite *L*, one has to solve Equations 6.5 and 6.6 numerically. Here, we will consider the simpler case of *x*-independent solutions, which occur for periodic boundary conditions or the Dirichlet boundary conditions in the large *L *limit (where boundary layers can be neglected). In this case, *q*(*x, t*) *→ q*(*t*), *p*(*x, t*) *→ p*(*t*) so that

(6.11) $$\frac{H}{L}=p\left(t\right)\left[-q\left(t\right)+F\left(W_{0}q\left(t\right)\right)+\frac{1}{2}p\left(t\right)g^{2}\left(q\left(t\right)\right)\right]\text{,}$$

and the optimal path is determined by the *x*-independent form of the Hamilton Equations 6.5 and 6.6:

(6.12) $$\dot{q}=-q+F\left(W_{0}q\right)+pg^{2}\left(q\right)$$

(6.13) $$\dot{p}=-p+W_{0}F^{\prime }\left(W_{0}q\right)p+p^{2}g\left(q\right)g^{\prime }\left(q\right).$$

Assuming that *F*(*q*) ~ *q *as *q *→ 0, we have

(6.14) $$p_{e}=-2\left(W_{0}-1\right)\underset{q\to 0}{\text{lim}}\frac{q}{g^{2}\left(q\right)}$$

**Figure 9:**
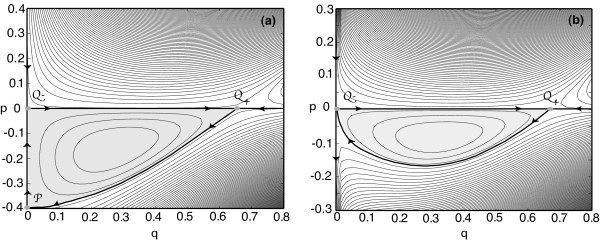
**Phase portrait of constant energy trajectories**. Phase portrait of constant energy trajectories for the Hamiltonian system given by Equations 6.12 and 6.13 with *F *(*q*) = tanh(*q*) and *g*(*q*) = *βq^s^*. Parameters are *W*_0 _= 1.2 and *β *= 1. Zero-energy trajectories are indicated by thick curves. The stable and unstable fixed points of the mean-field dynamics are denoted by $Q_{+}$ and $Q_{-}$. (**a**) *s *= 1/2: There exists a nonzero fluctuational fixed point P  that is connected to $Q_{+}$ via a zero-energy heteroclinic connection. The curve *Q*_+_*P *is the optimal path from the metastable state to the absorbing state. (**b**) *s *= 1/4: There is no longer a fluctuational fixed point P , so the optimal path is a direct heteroclinic connection between $Q_{+}$ and $Q_{-}$.

In Figure 9, we plot the various constant energy solutions of the Hamilton equations (Equations 6.12 and 6.13) for the differentiable rate function *F*(*q*) = tanh(*q*) and multiplicative noise factor *g*(*q*) = *βq^s ^*with *β *constant. The zero-energy trajectories are highlighted as thicker curves. Let us first consider the case *s *= 1/2 for which *p_e _*= 0.4*β*^-2 ^(see Figure 9a). As expected, one zero-energy curve is the line *p *= 0 along which Equation 6.12 reduces to the *x*-independent version of Equation 2.1. If the dynamics were restricted to the one-dimensional manifold *p *= 0, then the nonzero fixed point $Q_{+}=\left(q_{0},0\right)$ with *q*_0 _= *F*(*W*_0 _*q*_0_) would be stable. However, it becomes a saddle point of the full dynamics in the (*q, p*) plane, reflecting the fact that it is metastable when fluctuations are taken into account. A second zero-energy curve is the absorbing line *q *= 0 which includes two additional hyperbolic fixed points denoted by $Q_{-}=\left(0,0\right)$ and $P=\left(0,p_{e}\right)$ in Figure 9. $Q_{-}$ occurs at the intersection with the line *p *= 0 and corresponds to the unstable zero-activity state of the deterministic dynamics, whereas P  is associated with the effects of fluctuations. Moreover, there exists a third zero-energy curve, which includes a heteroclinic trajectory joining $Q_{-}$ at *t *= -*∞ *to the fluctuational fixed point P  at *t *= +*∞*. This heteroclinic trajectory represents the optimal (most probable) path linking the metastable fixed point to the absorbing boundary. The extinction time *τ_e _*is given by Equation 6.10 with $S_{0}/)L=\int_{Q_{+}}^{P}pdq\approx 0.15$, where the integral is evaluated along the heteroclinic trajectory from *Q*_+ _to *P *, which is equal to the area in the shaded regions of Figure 9a. For *s <*1/2, *p_e _*= 0 and the optimal path is a heteroclinic connection from $Q_{+}$ to $Q_{-}$. Hence, $S_{0}/)L=\int_{Q_{+}}^{Q_{-}}pdq\approx 0.05$.

Note that since the extinction time is exponentially large in the weak noise limit, it is very sensitive to the precise form of the action *S*_0 _and thus the Hamiltonian ℋ. This implies that when approximatingthe neural master equation of Buice et al. [46,54] by a Langevin equation in the form of Equation 5.1 with $\sigma \sim 1/)\sqrt{N}$, where *N *is the system size, the resulting Hamiltonian differs from that obtained directly from the master equation and can thus generate a poor estimate of the extinction time. This can be shown either by comparing the path integral representations of the generating functional for both stochastic processes or by comparing the WKB approximation of the master equation and corresponding Fokker-Planck equation. This particular issue is discussed elsewhere for neural field equations [55,56].

## 7 Discussion

In this paper, we have explored some of the consequences of having an unstable zero-activity state in a scalar neural field model. First, we considered invasive activity fronts propagating into the unstable zero state. These waves exhibit a behavior analogous to pulled fronts in reaction-diffusion systems, in which the wave speed is determined by the spreading velocity within the leading edge of the front. Consequently, front propagation is sensitive to perturbations in the leading edge, which we investigated within the context of spatial heterogeneities in the synaptic weights. We showed how time-dependent corrections to the wave speed could be estimated using a Hamilton-Jacobi theory of sharp interface dynamics combined with perturbation theory. Second, we considered a stochastic version of the neural field model, in which the zero-activity fixed point acts as an absorbing state. By constructing an integral representation of the neural Langevin equation, we showed how to estimate the time to extinction from a nontrivial steady state using a Hamiltonian formulation of large fluctuation paths in the weak noise limit.

It is clear from the results of this paper that neural fields with an unstable zero-activity state exhibit considerably different behaviors compared to models in which the zero state (or down state) is stable. Of course, one important question is whether or not real cortical networks ever exist in a regime where the down state is unstable. From a mathematical viewpoint, one has to choose a very specific form of the firing rate function *F *for such a state to occur (see Figure 1). Nevertheless, Buice and Cowan [46] have demonstrated that a stochastic neural field operating in a regime with an absorbing state belongs to the universality class of directed percolation models and consequently exhibits power law behavior suggestive of several measurements of spontaneous cortical activity *in vitro *and *in vivo *[47,48]. On the other hand, the existence of power law behavior is still controversial [61]. Irrespective of these issues, exploring the connections between nonlocal neural field equations and reaction-diffusion PDES is likely to be of continued mathematical interest.

## Competing interests

The author declares that they have no competing interests.
